# Effect of Waste Concrete Powder Content and Microwave Heating Parameters on the Properties of Porous Alkali-Activated Materials from Coal Gangue

**DOI:** 10.3390/ma17225670

**Published:** 2024-11-20

**Authors:** Vasilii Mischinenko, Andrey Vasilchenko, Georgy Lazorenko

**Affiliations:** 1 Technological Faculty, Platov South-Russian State Polytechnic University (NPI), Prosveshcheniya St., 132, Novocherkassk 346428, Russia; 2 Climate Center, Novosibirsk State University, Pirogov Street, 2, Novosibirsk 630090, Russia

**Keywords:** microwave foaming, geopolymer, alkali-activated materials, coal gangue, mine waste

## Abstract

The objective of this research is to fabricate waste-based alkali-activated foams with better properties in a quick time by using energy-efficient techniques such as microwave irradiation. The present study reports the effect of microwave heating parameters, including heating time and output power, on the properties of porous alkali-activated materials (AAMs) that use coal gangue (CG) as a precursor. The effects of concrete waste (CW) content (0–20 wt %) on the performance and microstructure of CG-based AAMs were investigated. Mechanical, thermal, and microstructural investigations were conducted to characterize the obtained materials. The experimental results indicate that the best characteristics of CG-based alkali-activated foams were achieved when microwave power and microwave heating time were 800 W and 10 min, respectively. The foams prepared by adding the waste concrete powder increased stability and showed lower bulk density and thermal conductivity. When the waste concrete powder content was 10 wt %, the CG-based alkali-activated foams showed the best overall performance. At the same time, the mechanical properties of the alkali-activated foams declined only slightly (~9%). The findings of this work provide a basis for further studies on improving the characteristics of CG-based alkali-activated foams due to the physical effect of a microwave field on fresh mortar without the use of a chemical foaming agent while reducing energy consumption in the production process.

## 1. Introduction

Meso- and macroporous inorganic materials obtained by alkaline activation of aluminosilicate raw materials (calcined kaolin [1], coal fly ash [2], slags of ferrous and non-ferrous metallurgy [3], etc. [4,5]) are of high practical interest due to their relatively high thermal stability, low density, and thermal conductivity. They are used in various fields of engineering as refractories [6], sorbents of heavy metals [7] and organic pollutants [8], and heat and sound insulating materials [9,10].

Coal gangue (CG) refers to the solid waste that is produced during excavation, mining, and coal washing processes. CG is a large tonnage waste whose formation can reach 15–20% of raw coal [11,12]. Long-term stacking of CG creates serious environmental problems. CG heaps and hills occupy significant areas, leading to land take and local habitat degradation [13,14]. The spontaneous combustion of piled CG leads to atmospheric pollution due to the emission of toxic and harmful gases, such as sulfur dioxides (SO_2_), nitrogen oxides (NO_x_), hydrogen sulfide (H_2_S), and carbon monoxide (CO), as well as smoke dust [15,16]. Because of weathering and leaching, heavy metals (e.g., Pb, Mo, Cr, Co, Cu, etc.) that are contained in CG are released, leading to soil and groundwater contamination [17,18,19].

CG has a predominantly aluminosilicate composition. The content of SiO_2_ and Al_2_O_3_ in CG in different habitats reaches 60–95% [11,12,20]. In addition, CG is characterized by a relatively high content of kaolinite (up to 67% [21]); its thermal transformation into the amorphous phase (metakaolin) makes it more reactive to alkalis. These features make CG attractive from the point of view of its use as a raw material for geopolymer and related alkali-activated materials [22,23,24,25].

CG-based foamed geopolymer materials were obtained by Su et al. [26], who used hydrogen peroxide as a foaming agent and sodium stearate as a stabling agent. From the experimental results, it was found that curing age has a significant effect on the mechanical properties of foams. The compressive strength and elastic modulus of CG-based geopolymer foams increased with water glass and H_2_O_2_. Meanwhile, the high sodium stearate content contributed to the opposite trend. Sitarz et al. [27] fabricated geopolymer foams based on thermally activated CG-containing kaolinite. Powdered aluminum and 36% hydrogen peroxide were employed as foaming agents for geopolymer mix preparation. Hydroxyethyl cellulose was added to enhance the foam stability. As the research has shown, stable geopolymer foams were obtained with porosities of 70% and 80% with two levels of powdered aluminum addition. Higher levels of foaming agents did not ensure higher porosity but only influenced its type, size, and pore distribution. CG-based lightweight geopolymers with the addition of metakaolin and waste glass were produced and characterized by Ziejewska et al. [28]. Hydrogen peroxide (35% solution in water) was used as a foaming agent. The obtained materials showed a compressive strength of 0.7–1.08 MPa, total porosity of 58.7–69.0%, and thermal conductivity of 0.101–0.113 W/mK (at 20–40 °C).

For the foaming of aluminosilicate alkali-activated materials, the interaction reactions of Al [29], Zn [30], or Si [31] metal powders are predominantly used, as well as concentrated hydrogen peroxide solution (H_2_O_2_) [32] and other chemical agents [33], in the alkaline environment of the solution mixture, accompanied by the release of gases (H_2_ or O_2_). Although the thermally cured alkali-activated foams obtained by this approach demonstrate relatively low thermal conductivity (<0.3 W/mK) and high total porosity (>50%), their significant disadvantage is their low compressive strength (<8.0 MPa) [34]. The method of physical foaming as a result of exposure to microwave radiation is an economical and energy-efficient alternative to the foaming of alkali-activated materials using chemical foaming agents. Compared with direct foaming or other foaming techniques [35,36], microwave heating has a significant advantage in low foaming and curing time. Recently, Li et al. obtained porous CG-based alkali-activated foams by microwave foaming for methylene blue removal [37] and pH buffering applications [38]. The obtained foams exhibited high total porosity (~85 vol%), as well as high-efficiency adsorption capacity and high pH buffering ability. At the same time, the compressive strength of the samples ranged from 0.46 MPa to 3.23 MPa. Although the authors used different microwave power settings, a systematic study of the influence of microwave heating parameters on the properties of porous alkali-activated materials from CG has not been conducted. In addition, the influence of associated raw materials on the foaming process and stability of the produced alkali-activated foams have not been investigated in previous studies.

The aim of this work is to optimize the fresh mortar mixes based on CG. The effect of concrete waste (CW) as a mineral admixture in enhancing the foam stability and hardened state properties of AAMs produced by a microwave foaming method is investigated. Scanning electron microscopy (SEM) and optical microscopy were employed to study the microstructure of the foams, while the mechanical properties were determined through compression strength testing. Then, the bulk density and thermal conductivity of the samples were investigated at several mixture parameters and preparation conditions. The purpose was to provide a new train of thought for the reuse of CG and concrete waste as raw materials in porous alkali-activated materials.

## 2. Materials and Methods

**Figure 1:**
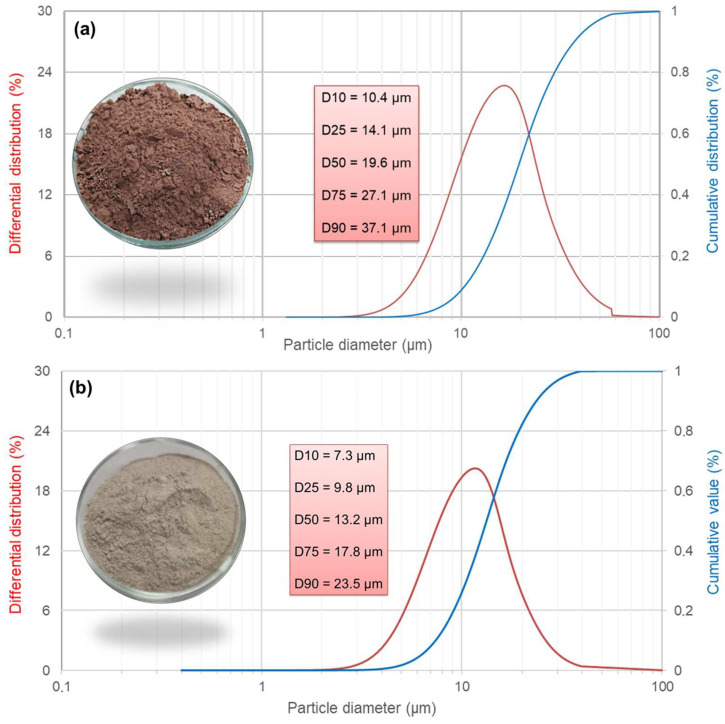
Particle size distribution of CG (**a**) and CW (**b**) specimens.

**Figure 2:**
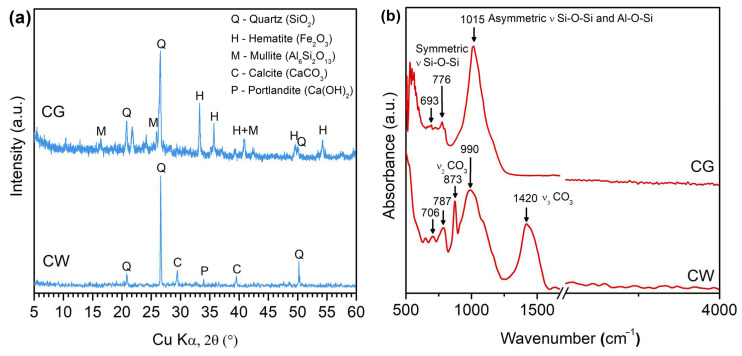
(**a**) XRD patterns and (**b**) FTIR spectra of CG and CW specimens.

Aluminosilicate precursor was pre-dried, crushed, and sieved through a sieve with a mesh size of 100 μm CG (Rostov region, Krasnosulinsky district, Gukovo). The SiO_2_, Al_2_O_3,_ and Fe_2_O_3_ oxide contents in the rock samples were 52.7%, 19.0%, and 17.4%, respectively. The particle size distribution (PSD) was identified by a Laska TD analyzer (Biomedical Systems LLC, Moscow, Russia). The PSD curve of the CG specimen is shown in Figure 1a. The mineralogical analysis and phase composition of ground samples were performed by X-ray diffraction analyses (XRD, D2 PHASER diffractometer, Bruker, Bremen, Germany) at a range of 5–58° with Cu Kα radiation (λ = 1.542 Å) and by attenuated total reflectance–Fourier transform infrared spectroscopy (ATR-FTIR, FT-801 Spectrometer, Simex LLC, Novosibirsk, Russia) in the region of 500–4000 cm^−1^. XRD patterns and FTIR data for the samples are reported in Figure 2. As observed, the CG used in this experiment was mainly composed of quartz (SiO_2_), hematite (Fe_2_O_3_), and mullite (Al_6_Si_2_O_13_) (Figure 2a). For the CG, the characteristic absorption bands were present at 1015, 776, and 693 cm^−1^ (Figure 2b). The absorption band at ~1015 cm^−1^ corresponded to the asymmetric Al–O–Si and Si–O–Si stretching vibration [39,40,41], while the peaks at 776 and 693 cm^−1^ were assigned to the symmetric Si–O stretching vibration [42].

Concrete waste passing through a 100 μm sieve was obtained from reclaimed crushed construction solid waste resources in Rostov-on-Don city. The PSD curve of the CW specimen is shown in Figure 1b. This waste concrete powder was then dried in an electric air-blast drying oven for 12 h at 105 °C. The CW was obtained by crushing and grinding, and the preparation process was similar to that of CG. The CW used in this experiment was mainly composed of quartz (SiO_2_), calcite (CaCO_3_), and portlandite (Ca(OH)_2_) (Figure 2a). For the CW, the band at 1420 cm^−1^ corresponded to the stretching vibration of the carbonate group, and the vibrational mode at 873 cm^−1^ was due to the out-of-plane bending of the carbonate group [43] (Figure 2b).

**Figure 3:**
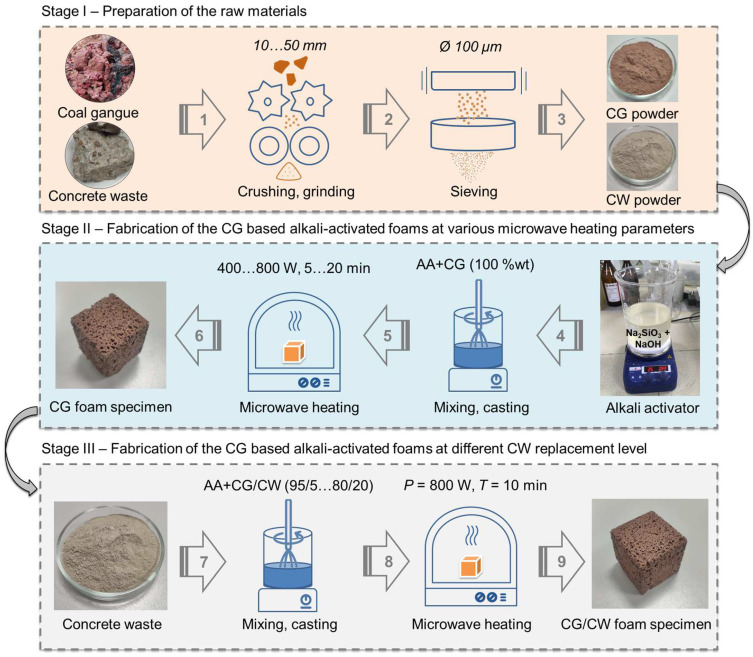
Manufacturing process of CG-based alkali-activated foams.

The alkaline activator solution was prepared by mixing 10 M of NaOH solution (analytical grade) and sodium waterglass (mass fraction of silicon dioxide, 30.2%; mass fraction of sodium oxide, 11.0%; silicate modulus, 2.89; density, 1.465 g/cm^3^) in a mass ratio 1:2.5. Both chemicals were purchased from Yug-Reaktiv Ltd. (Rostov-on-Don, Russia). The mixing process was carried out mechanically (150 rpm, 60 min), after which the activator solution was stored for 12 h before use. Mixing of solid fine precursor with alkaline activator solution in a mass ratio of 0.43 was carried out manually for 10 min until a homogeneous mortar mixture was obtained. Foaming and curing of the samples were carried out in silicone molds using an ultra-high-frequency heating chamber device with internal chamber dimensions of 240 × 354 × 358 mm and an operating frequency of 2450 MHz. The microwave output power (*P*) in the experiments conducted was 400, 600, 800, and 1000 W. The exposure time in the microwave heating chamber (*T*) was varied from 5 to 20 min in increments of 5 min. In the preparation of other samples, the microwave heating parameters were kept constant (800 W, 10 min), and the CGs were replaced with CW at the levels of 5%, 10%, 20%, and 15% by weight. A total of 11 groups of samples were prepared (Table 1). The variables of group CG/P400-T10, group CG/P600-T10, group CG/P800-T10, and group CG/P1000-T10 were set to microwave output power. The variables of group CG/P800-T5, group CG/P800-T15, and group CG/P800-T20 were set to the curing methods. The mortar mixtures with concrete waste were designated as follows: CG/CW5, CG/CW10, CG/CW15, and CG/CW20, indicating the addition of 5%, 10%, 15%, and 20% CW to the mix, respectively. A generalized manufacturing process of CG-based alkali-activated foams is shown in Figure 3.

The molded cube specimens with a face size of 50 mm were mechanically tested after 24 h to determine the compressive strength ($\sigma_{c}$) according to ASTM C109 using a dual-range E160N hydraulic test press (Matest, Treviolo, Italy) with an ultimate load of 15 and a 500 kN hydraulic loading actuator.

The average density ($\rho_{d}$) was determined as the ratio of the sample mass in an air-dry state to its total volume. Flat samples of 100 × 100 × 20 mm were applied to measure the heat transfer coefficient ($\lambda_{eff}$) under steady-state thermal conditions in accordance with GOST 7076 [44]. The tests were performed using the ITP-MG4 device (SKB Stroypribor, Chelyabinsk, Russia) in a working range of 0.02–1.5 W/mK. The tests were performed at room temperature and relative humidity of 22 ± 2 °C and 50 ± 10%, respectively. The final characteristics of cured mortar mixtures were taken as the arithmetic mean of the test results of five samples. The SEM images and EDX spectra of the sample were captured using a VEGA II LMU instrument (Tescan, Brno, Czech Republic) using an INCA ENERGY 450/XT system (Oxford Instruments, Oxford, UK).

## 3. Results and Discussion

### 3.1. Effect of Microwave Heating Parameters

**Figure 4:**
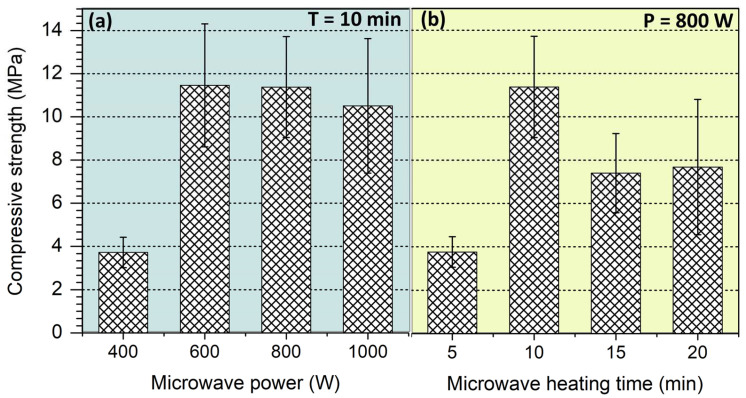
Effect of microwave power (**a**) and microwave heating time (**b**) on the compressive strength of CG-based alkali-activated foams.

Figure 4 shows the compressive strength of CG-based alkali-activated foams prepared with different microwave power and microwave heating times. The results of mechanical tests showed that the lowest compressive strength (3.7 MPa) is characterized by CG-based alkali-activated foam samples cured for 10 min in a microwave heating chamber with a microwave output power of 400 W (Figure 4a). Increasing the output power in the considered range leads to a sharp increase in compressive strength with values in the range of 10.5–11.5 MPa. The achieved mechanical characteristics of the CG-based alkali-activated foams exceeded those observed by researchers in previous studies [37,38,45]. Excessively long heating time (more than 10 min) can lead to the formation of microcracks or concentration of thermal stresses inside microwave-produced solid waste-derived foams [46], which negatively affects the strength (Figure 4b). A microwave chamber with an output power of more than 600 W can raise the temperature of the mortar mixture to 100 °C or higher in less than a minute. This sharp temperature increase facilitates a rapid reaction and the formation of an impermeable viscous binder. Free water evaporates, and it is held back by the viscous binder, forming a porous structure.

**Figure 5:**
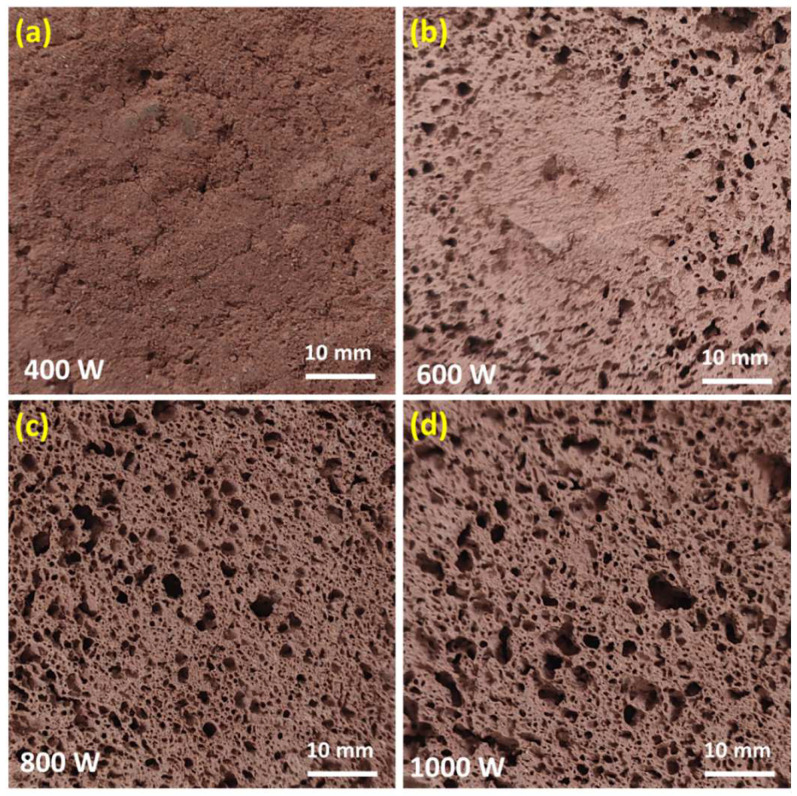
Cross-sections of the CG-based alkali-activated foam CG/P400-T10 (**a**), CG/P600-T10 (**b**), CG/P800-T10 (**c**), and CG/P1000-T10 (**d**).

**Figure 6:**
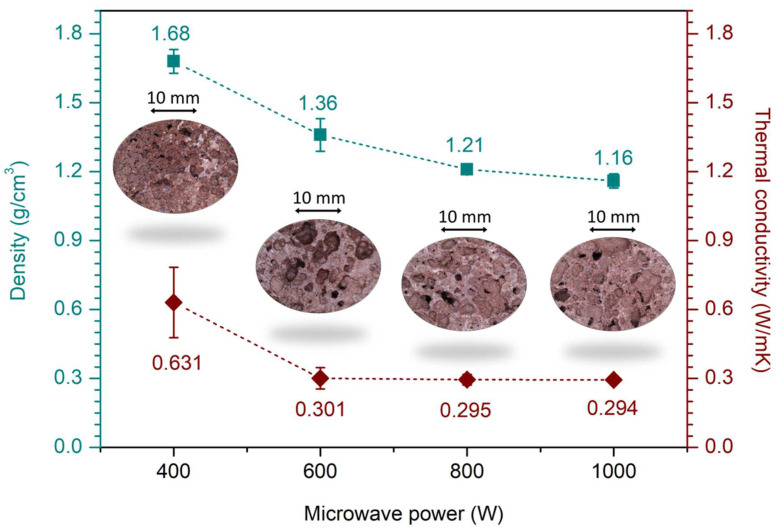
Density and thermal conductivity values of CG-based alkali-activated foams vs. microwave output power.

Figure 5 shows the details of the pore structure, while Figure 6 shows the density and thermal conductivity of CG-based alkali-activated foams. The experiment was conducted for a microwave heating time of 10 min. Microwave heating at low power values in the considered range provided a low degree of solidification and low porosity. Meanwhile, an increase in microwave power up to 800 W provided more uniform pore distribution and better pore shapes of CG-based alkali-activated foams (Figure 5c) due to more intensive water evaporation. Due to the cellular structure and low density, the samples had lower density and thermal conductivity at a microwave output power of 800 W and 1000 W (Figure 6).

### 3.2. Effect of Waste Concrete Powder Content

**Figure 7:**
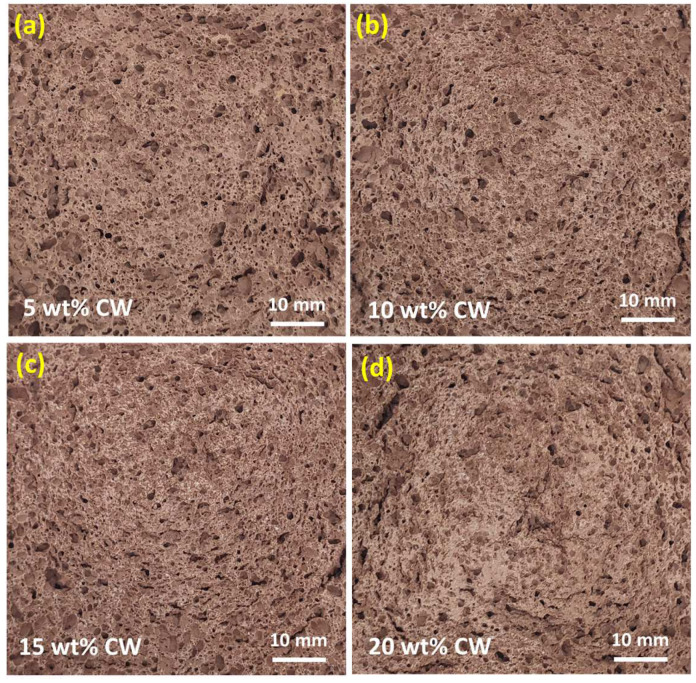
Cross sections of the CG-based alkali-activated foams with various dosages of CW: CG/CW5 (**a**), CG/CW10 (**b**), CG/CW15 (**c**), and CG/CW20 (**d**).

The effect of waste concrete powder on the bulk density is shown in Table 2. The incorporation of CW into the mix leads to a decrease in bulk densities of alkali-activated foams based on CG, the values of which for all four types of mixes were in the range of 1.19–1.26 g/cm^3^. The CG/CW5, CG/CW10, and CG/CW15 blends showed close bulk densities, while the $\rho_{d}\;$ of the CG/CW15 blends were lower. This may be attributed to the presence of more caverns and voids (Figure 7c) that are formed at the high content of waste concrete powder.

Figure 7 depicts cross sections of the CG-based alkali-activated foams with various dosages of CW, showing the distribution and pore size. The addition of waste concrete powder has a positive effect on the stability of the obtained alkali-activated foams. The change in porous structure is evident from the reduction in pore size. A similar trend was observed by Pasupathy et al. [47], who observed an increase in fine air voids when recycled concrete aggregates were added to aerated geopolymer concrete produced by mechanical foaming. Also, in foams with CW, a more uniform distribution of pores in the sample volume was observed compared with 100% CG-based foams (Figure 5), which have large, irregularly shaped pores. The increase in CW content did not lead to a significant difference in structure and porosity but affected the thermal insulation and mechanical properties.

Table 2 presents the results of the thermal conductivity of the CG-based alkali-activated foams with various dosages of CW. The obtained values are lower than those of 100% CG-based foams and range from 0.222 W/mK to 0.282 W/mK. Better insulation parameters were obtained for samples with 10 wt % waste concrete powder. At the same time, the mechanical properties of the alkali-activated foams declined only slightly (~9%) compared with the control group. This decrease in strength may be attributed to the high water absorption capacity of CW, leading to the loss of free water in mixtures containing CW. The optimal compressive strength was observed at a CW content of 10 wt %; in this case, the UCS value was 10.5 MPa. Thus, when the CW content was 10 wt %, the alkali-activated foams showed the best overall performance. The presence of Ca(OH)_2_ (derived from cement) contributed to the accelerated reaction and increase in the yield stresses of fresh mixtures, which may increase the stability of alkali-activated foams [47,48].

**Figure 8:**
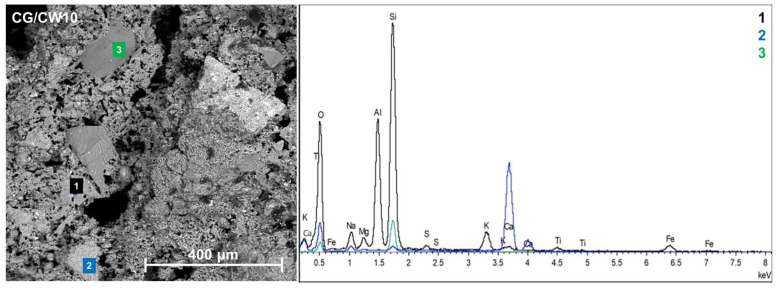
SEM-EDS micrograph of CG-based alkali-activated foam with 10 wt % waste concrete powder (CG/CW10).

CG-based alkali-activated foams with 10 wt % waste concrete powder exhibited a sponge-like morphology characterized by a porous structure containing many small, open, irregular pores (Figure 8). Compared with the specimens foamed without CW, the alkali-activated foams had a more uniform pore size structure. This behavior is in accordance with the observations in Section 3.2, in which the incorporation of waste concrete powder in the mixture has a positive effect on the compressive strength of CG-based alkali-activated foams. The EDX analysis shows the presence of energy peaks related to silicon, aluminum, calcium, oxygen, potassium, iron, and sulfur. The presence of Si, O, Al, and Na was expected from the CG and sodium-based alkaline activator, but the other elements were derived from the CW particles. Some unreacted CG and CW particles, such as quartz, which did not participate in geopolymerization in CG/CW10, filled the reaction products.

## 4. Conclusions

This work investigated the utilization of CG as a precursor to synthesize porous alkali-activated materials via microwave foaming. The influence of microwave heating parameters and waste concrete powder content on the bulk density, mechanical properties, microstructure, and thermal conductivity of the CG-based alkali-activated foams were investigated. The main conclusions are drawn as follows:

(1) The optimal conditions were a microwave heating time of 10 min and microwave power of 800 W. At the optimal conditions, compressive strength was estimated to be 11.5 MPa.
(2) The microstructure characterization of CG-based alkali-activated foams illustrated that samples foamed at high microwave power have a more uniform pore distribution and better pore shapes.
(3) The foams prepared by adding the CW have increased stability and showed lower bulk density and thermal conductivity. The waste concrete powder helps trap the foam, refine the pore size, and homogenize the pore size range.
(4) When the waste concrete powder content reached 10 wt %, alkali-activated foams’ thermal conductivity decreased from 0.295 W/mK to 0.222 W/mK. At the same time, the mechanical properties of the alkali-activated foams decreased only slightly (~9%).

Thus, the microwave foaming method can be used to obtain porous alkali-activated materials based on coal mining waste without the use of chemical pore formers, resulting in a simultaneous reduction of energy consumption in production processes. Further studies could investigate the effect of other parameters, such as the type and mixture proportion of the alkali activator, on the fresh and hardened state properties of the CG-based alkali-activated foams. Additionally, a systematic analysis of environmental impact should be considered in future work, including a life cycle assessment (LCA), in order to find a reasonable and economical design. There is also a need for further research to characterize the functional properties of CG-based alkali-activated foams.

## Figures and Tables

**Table 1:** Mix parameters and proportions of the CG-based alkali-activated foams.

Mix Name	NaOH Concentration (mol/L)	Solid-to-Liquid Ratio (by Mass)	CG Content (wt.%)	CW Content (wt.%)	Microwave Output Power (W)	Microwave Heating Time (min)
CG/P400-T10	10	0.43	100	–	400	10
CG/P600-T10	10	0.43	100	–	600	10
CG/P800-T10	10	0.43	100	–	800	10
CG/P1000-T10	10	0.43	100	–	1000	10
CG/P800-T5	10	0.43	100	–	800	5
CG/P800-T15	10	0.43	100	–	800	15
CG/P800-T20	10	0.43	100	–	800	20
CG/CW5	10	0.43	95	5	800	10
CG/CW10	10	0.43	90	10	800	10
CG/CW15	10	0.43	85	15	800	10
CG/CW20	10	0.43	80	20	800	10

**Table 2:** Bulk density, compressive strength, and thermal conductivity values of CG-based alkali-activated foams with various dosages of CW.

Mix Name	Bulk Density (g/cm^3^)	Δ	Compressive Strength (MPa)	Δ	Thermal Conductivity (W/mK)	Δ
CG/CW5	1.25	±0.01	8.3	±0.9	0.255	±0.047
CG/CW10	1.26	±0.02	10.5	±0.4	0.222	±0.042
CG/CW15	1.26	±0.07	8.5	±0.7	0.278	±0.017
CG/CW20	1.19	±0.02	7.2	±0.3	0.282	±0.011

## Data Availability

The raw data supporting the conclusions of this article will be made available by the authors on request.
